# Molecular Characterization of Newcastle Disease Virus from Backyard Poultry Farms and Live Bird Markets in Kenya

**DOI:** 10.1155/2018/2368597

**Published:** 2018-08-05

**Authors:** Irene N. Ogali, Lucy W. Wamuyu, Jacqueline K. Lichoti, Erick O. Mungube, Bernard Agwanda, Sheila C. Ommeh

**Affiliations:** ^1^Veterinary Research Institute, Kenya Agriculture and Livestock Research Organization, P.O. Box 32-00902, Kikuyu, Kenya; ^2^Institute of Biotechnology Research, Jomo Kenyatta University of Agriculture and Technology, P.O. Box 62000-00200, Nairobi, Kenya; ^3^Directorate of Veterinary Services, State Department of Livestock, Ministry of Agriculture Livestock and Fisheries, Kangemi, Nairobi 00625, Kenya; ^4^Department of Zoology, National Museums of Kenya, P.O. Box 40658-00100, Nairobi, Kenya

## Abstract

Newcastle disease (ND) is a serious disease of poultry that causes significant economic losses. Despite rampant ND outbreaks that occur annually in Kenya, the information about the NDV circulating in Kenya is still scarce. We report the first countrywide study of NDV in Kenya. Our study is aimed at evaluating the genetic characteristics of Newcastle disease viruses obtained from backyard poultry in farms and live bird markets in different regions of Kenya. We sequenced and analyzed fusion (F) protein gene, including the cleavage site, of the obtained viruses. We aligned and compared study sequences with representative NDV of different genotypes from GenBank. The fusion protein cleavage site of all the study sequences had the motif 112RRQKRFV118 indicating their velogenic nature. Phylogenetic analysis revealed that the NDV from various sites in Kenya was highly similar genetically and that it clustered together with NDV of genotype V. The study samples were 96% similar to previous Ugandan and Kenyan viruses grouped in subgenotype Vd This study points to possible circulation of NDV of similar genetic characteristics between backyard poultry farms and live bird markets in Kenya. The study also suggests the possible spread of velogenic NDV between Kenya and Uganda possibly through cross-border live bird trade. Our study provides baseline information on the genetic characteristics of NDV circulating in the Kenyan poultry population. This highlights the need for the ND control programmes to place more stringent measures on cross-border trade of live bird markets and poultry products to prevent the introduction of new strains of NDV that would otherwise be more difficult to control.

## 1. Introduction

Newcastle disease (ND) is a highly contagious and fatal disease of poultry which is notifiable to the World Organization for Animal Health (OIE) [1]. The disease is present worldwide and affects many domesticated and wild bird species [2]. It is caused by Newcastle disease virus (NDV) which is classified under the genus *Avulavirus* within the family Paramyxoviridae [3]. NDV is an enveloped, single-stranded negative-sense RNA virus whose genome is approximately 15 kb. Its genome has six open reading frames (ORFs) which encode for six major structural proteins, namely, nucleoprotein (NP), phosphoprotein (P), matrix protein (M), fusion protein (F), hemagglutinin-neuraminidase (HN), and the RNA-dependent RNA polymerase (L) [4]. NDV also has two nonstructural proteins, W and V, from differential initiation or transcriptional editing of the P gene mRNA [5].

NDV strains produce a range of pathogenic outcomes in poultry. Viruses of low pathogenicity or lentogenic result in subclinical disease, whereas those of moderate pathogenicity or mesogenic generally causes clinical signs of disease, but typically result in nonlethal outcomes in chickens. Viruses with high pathogenicity or velogenic cause serious disease and mortality among affected birds [2]. The cleavability of the fusion protein precursor (F_0_) and the presence of a number of basic residues in the fusion protein cleavage site are major determinants for NDV pathogenicity [6]. Velogenic NDV has a multiple basic amino acid sequence: ^112^R/K-R-Q-K/R-R^116^ at C terminus of the F2 protein and F (phenylalanine) on residue 117, whereas lentogenic and mesogenic viruses have a monobasic amino acid sequence: ^112^G/E-K/R-Q-G/E^116^ and L (leucine) on residue 117 [7].

The fusion protein gene displays greater antigenic variation than the internal genes and is important in studying closely related virus populations to allow deduction of the evolutionary hypothesis. Genome size analysis, as well as sequences of F and L genes, has revealed two distinct categories of NDV: Class I or Class II. Class I viruses are mostly lentogenic and have been recovered primarily from waterfowl, shorebirds, and domestic poultry [8]. Class II consists mainly of velogenic viruses isolated from domestic poultry and wild birds that have been grouped into eighteen genotypes [9–11]. The latter clade of the virus is responsible for most of the ND outbreaks that cause huge economic losses to the poultry industry globally [12]. The NDV genome is highly diverse and continues to evolve [5]. In Africa, genetic studies have isolated velogenic NDVs from sick and healthy poultry [13, 14]. In the recent years, there has been an emergence of novel genotypes of NDV, the bulk of which have been isolated from African countries [15].

In Kenya, like other African countries, ND causes devastating losses to the poultry industry [16]. In backyard poultry, the disease is endemic but recurs as frequent epidemic outbreaks with high mortality and thus affects the livelihoods of poor rural households who depend on poultry for food and income [17]. In Kenya, backyard poultry has been implicated in the introduction and spread of velogenic NDV strains [18]. Backyard poultry management favors the existence and spread of diverse NDV strains by allowing free interaction of different poultry species and wild birds as well as the frequent introduction of birds from markets [19]. Despite the potential threat posed by backyard poultry in the evolution of NDV, there is limited information about the genetic profile of NDVs circulating in backyard poultry in Kenya. The present study analyzed the fusion gene of NDV obtained from poultry in live bird markets (LBMs) and backyard poultry farms (BPFs) in Kenya. The fusion protein gene sequence from the study samples was compared with NDV of known genotypes present in GenBank.

## 2. Materials and Methods

### 2.1. Study Area

A cross-sectional study was conducted between November 2015 and March 2016 in backyard flocks and live bird markets (LBMs) in Kenya. The study was undertaken in rural backyard poultry farms (BPFs) in Western Highlands, Lake Victoria Basin, and Coastal regions of Kenya. These three regions were chosen purposively due to their high population density of backyard poultry [20]. Five wards were randomly selected from each of the three regions as follows: Western Highlands (Chwele, Cheptais, Kimilili, Kabuchai, and Malaba), Lake Victoria Basin (Bunyala, Ageng'a, Ugenya, Chakol, and Amukura), and Coastal regions (Kakoneni, Likoni, Mtepeni, Dabaso, and Mkomani). In addition, twenty live bird markets were sampled from five regions, namely, Western Highlands, Lake Victoria Basin, Coast, Nairobi, and Eastern regions. The sampled markets included the following: Western Highlands (Chwele, Bungoma, Kitale, Kericho, Kakamega, and Bomet), Lake Victoria Basin (Bumala, Kisumu, Homa Bay, and Migori), Coast (Majengo, Marikiti, and Kilifi), Eastern (Meru and Makueni), and Nairobi metropolitan (Burma, Kibra, Kawangware, Machakos, and Kitengela).

### 2.2. Sample Size and Selection of Study Birds

A total of 1,224 birds were sampled from 225 BPFs. The minimum sample size from backyard farms for each of the three zones was calculated based on the following formula [21]:

(1) $$\begin{array}{c} n=\frac{Z^{2}P\left(1-P\right)}{d^{2}}, \end{array}$$

where *n* = sample size, *Z* = *Z*-statistic for a level of confidence, *P* = expected prevalence, and *d* = precision prevalence of ND, estimated to be 20% from a previous study [22]; a confidence level of 95% and a precision of 5% were used. From each of the selected wards, fifteen poultry keepers who had a history of not vaccinating their flock were randomly selected. From each farm, 4 adult birds were sampled. From farms with different species of poultry, 2 birds of each species were sampled.

During sampling, each selected bird was physically examined for any signs suggestive of ND infection (diarrhoea, ocular and nasal discharge, respiratory distress, nervous signs, or sudden death). During sampling, freshly dead birds (not more than 12 hours) were collected for a postmortem examination at the nearest Veterinary Investigative Laboratories (VILs). For such birds, tissues were collected. Tissue samples from an individual bird were pooled. In total, we collected samples from 922 chickens (including tissues from 96 chickens, each from a different farm), 136 ducks, and 74 turkeys from BPFs.  Table 1 shows the number and species of birds sampled per region.

In live bird markets, a total of 482 birds were sampled from 124 traders. The number of birds sampled in each market was calculated assuming an average market size of 30 birds, a minimum expected prevalence of 5%, and a confidence interval of 95%. Twenty LBMs were selected from the five zones. In the LBMs, five sellers were selected randomly, and four birds were randomly selected per seller for sampling. However, in 7 live bird markets that had high bird turnover, we sampled 7 traders each. This included Kisumu, Kericho, Majengo, Burma, Meru, and Chwele markets. In selection of birds to be sampled in LBMs, information on the source of the birds and the type of management was sought. When it was established that the birds had been vaccinated, they were excluded from the study. In total, we sampled 454 chickens (including tissues from 33 chickens) and 28 ducks. The number and species of birds sampled in LBMs are shown in Table 1.

### 2.3. Sampling of Birds

Tracheal and cloacal swabs were collected from each individual bird that was selected for sampling in BPFs and LBMs except for freshly dead birds (not more than 12 hours). From the latter, tissue samples were collected after a thorough postmortem examination following laid out procedures [23]. Tissue samples collected included the lungs, trachea, liver, spleen, proventriculus, gastrointestinal tract, and brain tissue. Different tissues were pooled per individual bird.

Each sample was collected in 1000 *μ*l of RNAlater® (Thermo Fisher Scientific, Waltham, Massachusetts, USA). The collected samples were transported in a cool box and stored at −20°C, and RNA extraction was carried out within 72 hours of sample collection.

### 2.4. Viral RNA Extraction

In the laboratory, tracheal and cloacal swab samples from each backyard farm were pooled separately by poultry species to generate a total of 1128 pairs of swab samples. On the contrary, swab samples from individual birds sampled in markets were processed separately, resulting in 449 pairs of swab samples from markets. One hundred twenty-nine (129) pools of tissue samples from 129 chickens (96 from farms and 33 from LBMs) were also processed separately.

We extracted viral RNA using the Trizol LS reagent (Invitrogen, Carlsbad, CA, USA). The cryotubes with swabs were vortexed vigorously for 2 minutes and then centrifuged at 300 ×g for 10 minutes in a prechilled centrifuge at 4°C, and the supernatant was aliquoted in 500 *µ*l volumes and transferred into sterile 2000 *µ*l cryotubes. To one aliquot, 1000 *µ*l of the Trizol reagent was added. RNA was extracted using the Trizol LS reagent according to the manufacturer's instructions.

Tissue samples were crushed using a sterile mortar and a pestle and placed in a sterile 2000 *µ*l cryotube into which 1000 *µ*l of the Trizol reagent was added, and RNA was extracted using the Trizol LS reagent according to the manufacturer's instructions. We resuspended the RNA in DEPC-treated water. The quantity of the extracted RNA was determined using a NanoDrop® ND-1000 spectrophotometer (Thermo Fisher Scientific, Waltham, Massachusetts, USA), and the integrity of RNA was visualized by electrophoresis in a 1.2% formaldehyde-agarose gel stained with GelRed. The RNA was stored at −80°C in aliquots of 10 *µ*l until use.

### 2.5. Fusion Gene Amplification and Sequencing

Comple-mentary DNA was synthesized using 5 *µ*l of eluted RNA with random hexanucleotides and the SuperScript® III (M-MLV) reverse transcriptase of the First-Strand cDNA Synthesis Kit (Invitrogen, Carlsbad, CA, USA).

The samples were tested by conventional PCR amplification of the fusion gene using degenerate primers previously published by Liu et al. which target the cleavage site of the fusion protein gene [24]. The two degenerate primers, 5′-ATG GGC (C/T) CC AGA C (C/T)CT TCT AC-3′ (sense, from nt 47–66 of the F gene) and 5′-CTG CCA CTG CTA GTT GTG ATA ATC C-3′ (antisense, from nt 557–581 of the F gene), amplified a 535 bp fragment of the fusion protein gene. Primer blast analysis [25] of the primers established that the primers could detect a broad range of ND viruses. The fusion gene region amplified by the primers has been used previously to detect a wide range of NDVs [26]. The PCR was performed using Taq DNA polymerase and 5 *µ*l of cDNA with the cycling parameters starting with a denaturation step at 95°C for 3 min and subsequent parameters as described by Liu et al.

Amplification of the full fusion gene (1780 bp including the fusion gene start) was carried out successfully for one positive sample. To achieve this, a seminested PCR was done using Platinum Taq DNA polymerase (Platinum® *Taq* DNA polymerase High Fidelity) and previously published primers: F1 for 5′-ACGGGTAGAAGATTCTG-3′, F2 for 5′GTTGACTAAGTTAGGTG 3′, and F3 5′-CTCTCCGAATTGACAGAC-3′ [27]. Primers F1 and F2 were used in the first PCR and primers F2 and F3 were used in the second PCR to amplify the full fusion gene. The conditions for the two PCRs were similar and included an initial denaturation at 95°C for 2 min and then 35 cycles at 95°C for 30 s, 55°C for 1 min, and 72°C for 1 min and a further extension at 72°C for 10 min.

The PCR products were purified using the QIAquick PCR Purification Kit (Qiagen) and were sent to Macrogen® Inc (South Korea) for sequencing on an Applied Biosystems 3100 automated DNA sequencer using dye terminator cycle sequencing chemistry (Applied Biosystems, Foster City, CA, USA). Sequencing was done in both directions using the same primers used for PCRs. Identical sequences were not deposited in GenBank, instead they were represented by one sequence. Partial F gene sequences were deposited in GenBank with accession numbers *KY007043* to *KY007063*. The full coding nucleotide sequence of the fusion gene generated was deposited in GenBank under accession number *MG988405*.

### 2.6. Phylogenetic Analysis

We assembled sequences and removed those of low quality using Chromas Lite version 2.6. Unique sequences were identified using DNA sequence polymorphism (DnaSP v5.10) [28]. These unique sequences were compared with reference sequences from other parts of the world, selected to represent the NDV genotypes reported to date. At least three sequences representing each genotype were used in the analysis. Other sequences similar to the study sequences in GenBank obtained using the BlastN algorithm [29] were also included in the analysis (the reference sequences from GenBank used in this study and their accession numbers are in Supplementary Table S1). Multiple alignment and comparison of the study sequences and GenBank references were performed using MUSCLE v3.8.31 [30]. Phylogenetic and molecular evolutionary analyses were conducted using MEGA (Molecular Evolutionary Genetics Analysis) version 6.0 [31]. We constructed phylogenetic trees using the maximum likelihood (ML) method and estimated the tree using the best-fit general time-reversible (GTR) model of nucleotide substitution with gamma-distributed rate variation among sites. We employed a bootstrap resampling process (1000 replications) to assess the robustness of individual nodes of phylogeny.

The study also estimated the percent (%) similarity and the mean evolutionary distance between the complete fusion gene of the study sample and representative viruses of genotypes V and II.

## 3. Results

**Figure 1:**
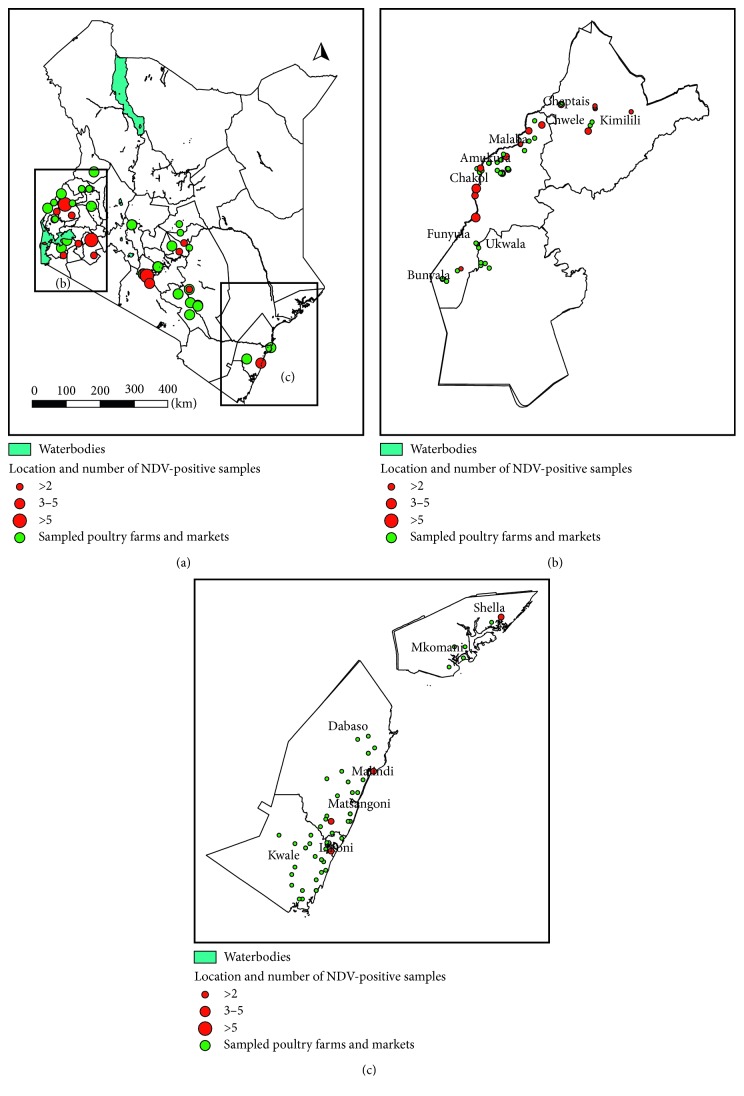
Geographical location of NDV-positive backyard poultry farms and live bird markets in Kenya, from November 2014 to March 2016: (a) a map of Kenya showing the location of sampled and NDV-positive live bird markets; (b) a map of the study region in Western Kenya showing the location of sampled and NDV-positive backyard poultry farms; (c) a map of Coastal region showing the location of sampled and NDV-positive backyard poultry farms.

Overall, 1,224 poultry samples (1128 swabs and 96 tissue samples) from poultry flocks and 482 samples (449 swabs and 33 tissue samples) from live bird markets were tested. Using the partial F gene assay, 2.7% (33/1224) and 10.8% (52/482) of samples from BPFs and LBMs, respectively, tested to be positive.  Figure 1 shows the location of NDV-positive samples obtained from BPFs and LBMs across different regions of Kenya. The positive samples were concentrated along the Kenya-Uganda border but were also found in different other regions of Kenya. All positive samples were obtained from chicken except one duck sample from a poultry farm in Western Highlands that tested to be positive. From this farm, the sample from chicken also tested to be positive for NDV. A higher proportion of tissue samples tested to be positive: 15.8% (15/96) and 48.5% (16/33), compared to 1.6% (18/1128) and 8% (36/449) of swab samples from farms and LBMs, respectively.

Table 2 shows the bird-level detection of NDV in BPFs and LBMs in different regions. Regional differences were observed in the detection of NDV. Nairobi LBMs and Lake Victoria Basin BPFs had significantly higher NDV detection. In both farms and LBMs, NDV detection was higher in birds that exhibited clinical symptoms; NDV was detected in 7.4% (23/312) and 39% (23/59) of clinically sick birds in farms and LBMs, respectively, compared to 1.1% (10/912) and 6.9% (29/423) of NDV detection in healthy birds from farms and LBMs, respectively.

### 3.1. Proteolytic Cleavage Site of the Fusion Protein

A total of 57 partial fusion gene sequences were successfully obtained; this included 31 and 26 sequences from LBMs and BPFs, respectively. Of these, 22 nonidentical sequences were identified and used for analysis. Five of the unique sequences were found commonly in samples obtained from both LBMs and BPFs, seven of the sequences were obtained from BPFs, while ten were obtained from LBMs. The 57 sequences were obtained from chicken samples and one duck sample. The sequence obtained from the duck sample was identical to the sequence from a chicken sample (sample KE0697/2015 (accession number *KY007050*) obtained from the same farm). The complete fusion gene coding sequence was sequenced from one sample among 22 which had a unique partial fusion gene sequence (sample KE1007/2016: *MG988405*).

Analysis of the amino acid sequence of the fusion protein gene of the obtained NDV exhibited similar properties. We compared the deduced amino acid sequences with other strains of NDV. The sequence analysis of the amino acid of the protease cleavage site revealed that the F1 protein of all the study sequences contains a phenylalanine (F) on residue 117 on the N-terminus and four basic amino acids in the motif ^112^GRRQKR^116^ ∗ F^117^, while one sequence KY007062 harbours the motif ^112^GRRQRR^116^ ∗ F^117^. This indicates that the study samples are velogenic.

### 3.2. Diversity and Phylogenetic Relationship of NDV from Kenya

**Figure 2:**
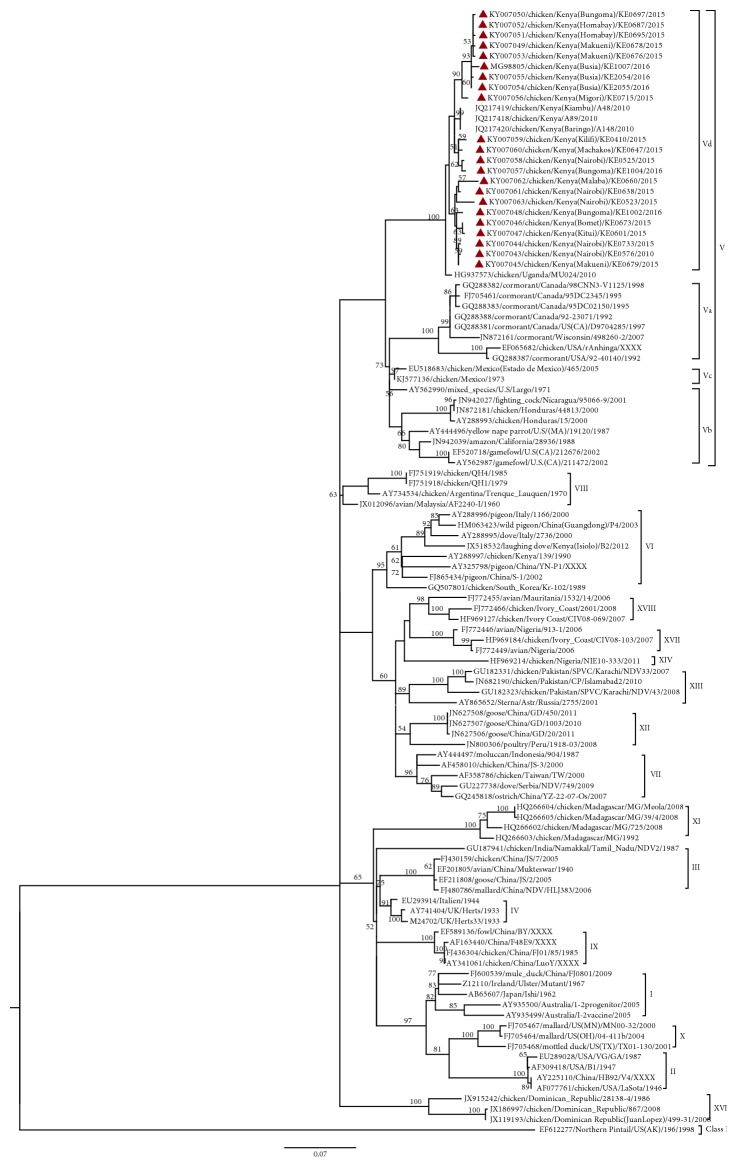
Phylogenetic tree of partial fusion (F) gene nucleotide sequences of the Newcastle disease virus- (NDV-) positive study samples (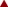) and GenBank references. The percentage of replicate trees in which the associated taxa clustered together in the bootstrap test (1000 replicates) is shown next to the branches (only >50% is shown). The evolutionary history was inferred by using the maximum likelihood method based on the Kimura 2-parameter model. The gamma correction for rate heterogeneity was 0.4292. The analysis involved 111 nucleotide sequences. The tree is rooted using the NDV sequence belonging to Class I. There were a total of 521 positions in the final dataset. Evolutionary analyses were conducted in MEGA 6 [31].

We illustrate the relationship between positive samples obtained in the study and other NDVs available in GenBank, using a phylogenetic tree of the partial F gene sequence of the 22 unique sequences obtained in the study and the other GenBank sequences of NDV (Figure 2). From the tree, all the 22 study sequences were grouped together with representative strains of genotype V. Within genotype V, the study samples were genetically more closely identical (Figure 2) to previous Kenyan (*JQ217418*, *JQ217419*, and *JQ217420*) and Ugandan (*HG937573*) sequences. The study sequences together with previous Ugandan and Kenyan strains formed a distinct clade branching with 100% bootstrap value at the defining node from other subgenotypes of genotype V (Figure 2) including genotype V viruses from Europe (Supplementary Figure S2).

**Figure 3:**
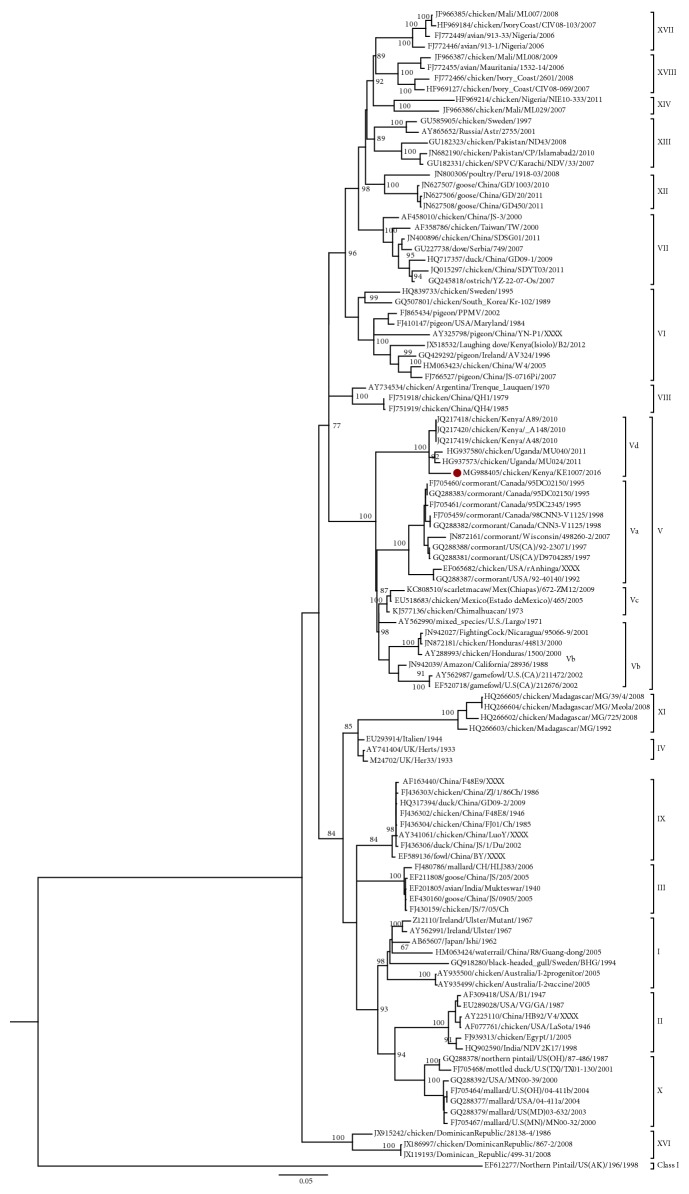
Phylogenetic tree of nucleotide sequence of the complete fusion (F) gene of Newcastle disease virus (NDV) obtained in this study (marked 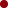) and reference sequences from GenBank. The percentage of replicate trees in which the associated taxa clustered together in the bootstrap test (1000 replicates) is shown next to the branches (only >50% is shown). The evolutionary history was inferred by using the maximum likelihood method based on the Tamura Nei model [31]. The gamma correction for rate heterogeneity was 0.4141. The analysis involved 109 nucleotide sequences. The tree is rooted using the NDV sequence belonging to Class I. There were a total of 1662 positions in the final dataset. The analyses were conducted in MEGA 6.

To confirm the phylogenetic classification of the study sequences, we constructed a phylogenetic tree of one complete fusion gene coding sequence obtained in the study sample and other NDV sequences present in GenBank (Figure 3). The tree grouped NDVs of Class II into two groups: one consisting of the older genotypes I–IV and related newer genotypes IX and XI and another group consisting of the newer genotypes V–XVIII. Similar to the partial F gene sequences, the complete F gene sequence obtained in the study (sample KE1007/2016) was grouped with other sequences of genotype V but more closely to previous Kenyan and Ugandan strains. We determined the percentage identity of the obtained complete fusion gene nucleotide sequence compared to NDV of genotype V representing the various subgenotypes Vd (*HG937573*), Vb (*AY288993*), Vc (*KC808510*), and Va (*GQ288382*) and the vaccine strain LaSota (*AF077761*) (Table 3). The results showed that the study virus was more identical to Ugandan strain (*HG937573*) of subgenotype Vd (97%) than to other viruses of subgenotype V, which were 91% (Vc strain *KC808510*), 90% (Vb strain *AY288993*), and 89% (Va *GQ288382*) similar to the study sample. The study sequence was 83% similar to the LaSota vaccine strain (*AF077761*) commonly used in Kenya. We also determined the mean evolutionary distance between the complete fusion gene nucleotide sequence obtained in the study and reference sequences of subgenotypes of genotype V (Table 3). The mean evolutionary distances between the study sequences and subgenotype Vd, Vb, Vc, and Va sequences were 3.1%, 12.6%, 10.7%, and 14.2%, respectively. The evolutionary distance between the study fusion gene sequence and the genotype II sequences was 23.5%. The mean nucleotide distance between subgenotype Vd and other subgenotypes of genotype V ranged from 9.5% to 13%. An analysis of the amino acid sequence of the fusion gene of our study sample revealed six unique amino acid substitutions: methionine at position 28, glutamic acid at position 104, lysine at position 146, valine at position 299, threonine at position 517, and alanine at position 550.

## 4. Discussion

The present study represents the first countrywide genetic study of prevailing Newcastle disease viruses in Kenyan backyard poultry. Analysis of the NDV-positive samples showed the existence of genetically closely related NDVs in the sampled live bird markets (LBMs) and backyard poultry farms (BPFs). This is an indication of the continuous movement and exchange of viruses between BPFs and LBMs in different parts of the country. LBMs in Kenya consist of poor biosecurity practices where birds from different sources interact [33], and this could be responsible for the higher number of positives detected in LBMs compared to BPFs. The proportion of positive samples in BPFs was much lower than that found previously by Chaka et al. [34] in Ethiopia who used an L gene real-time reverse transcription PCR (rtRT-PCR). In our study, we used the partial fusion gene assay to detect NDV. The partial F gene has been used in the previous study to detect a wide range of NDVs [26]. In addition to the partial F gene assay, the study also utilized a partial L gene NDV reverse transcription PCR (RT-PCR) assay to test all the samples and obtain L gene sequences. The L gene assay could detect both Class I and II viruses. The results of both the L gene and F gene assays were well correlated. These findings can be made available upon request.

Analysis of the sequence of the positive samples indicated their similarity to the viruses in GenBank classified under genotype V. Genotype V alongside genotypes VI, VII, and VIII as well as novel genotypes VI–XVIII of NDV has been responsible for recent outbreaks worldwide [12]. In particular, genotype V viruses have been associated with ND outbreaks that occurred in various parts of the world from the 1970s to the 1980s. Genotype V emerged in South America and Central America in the 1970s [35] and was linked to outbreaks in Europe in that same period [36]. These viruses also caused outbreaks in North America and other parts of the world between the 1970s and 1980s [37, 38]. It is possible that genotype V NDV could have been introduced in Kenya during this period and continues to survive in the backyard poultry. However, the study findings indicate that the NDV in Kenyan backyard poultry seems to form a separate cluster that is genetically unique from other viruses of genotype V. This points to a possibility of independent evolution of NDV in the local bird population in Kenya. Genotype V is classified into three subgenotypes: Va, Vb, and Vc [34, 38]. Subgenotype Va includes viruses isolated from cormorant species in North America [38] and Vb includes viruses isolated mainly from poultry in Mexico and Central America [39, 40]. In Europe, both subgenotypes Va [41] and Vb [42] have been detected. Subgenotype Vc composed of viruses isolated in Mexico from 2004 to 2010 is thought to have evolved from subgenotype Vb [35].

The study found particular concentration of positive NDV samples in BPFs and LBMs at the Kenya-Uganda borders. Through analysis of both the partial and complete fusion gene sequences, the study established that the NDV-positive samples along the border and those from different regions of Kenya were velogenic, exhibiting a higher similarity to previous viruses from Uganda. Together with Ugandan and previous Kenyan viruses, our study samples formed a distinct clade from other subgenotypes of genotype V. To date, this strain of viruses unique to Kenya and Uganda has not been found in any other country. This suggests exchange of NDVs across the Kenya-Uganda border possibly through uncontrolled cross-border live bird trade. A previous study suggested that Ugandan and Kenyan viruses of NDV could fall into a new subgenotype Vd which could have a possible independent evolution from the other genotype V NDV [32]. Our study found an average nucleotide distance of 11.5%, 9.8%, and 13% between subgenotype Vd and Vb, Vc, and Va, respectively. This suggests that Vd with which the study sample clusters belongs to an additional subgenotype within genotype V. A new subgenotype is assigned if it has viruses collected from more than four independent epidemiological studies, has a high bootstrap value (>60%) at the defining node on the phylogenetic tree, and has an average nucleotide distance between groups of 3% to 10%. In this study, we used samples collected from three independent epidemiological studies. Although NDV strains from more studies are required to support this, the study findings give evidence of the need for probable inclusion of new subgenotype Vd within genotype V.

This study compared the similarity of the fusion protein gene of study samples to LaSota strain which is the vaccine widely used in Kenya for ND control and found a similarity of 83%. This value is not an indicator of the level of protection of the vaccine against Kenyan NDV which can only be evaluated in detailed efficacy trial of the current vaccine against the Kenyan field strains of NDV.

All the positive samples in this study contained velogenic NDV, even from seemingly healthy birds. The study sequences were all obtained from chicken samples; however, one duck sample had a fusion gene sequence identical to sequences obtained from chicken in the same farm. Moreover, the cleavage site of the sequence from the duck sample was also indicative of presence of a velogenic virus. This points to continuous circulation of velogenic NDV between backyard poultry species in Kenya. The study, however, was limited to genetic characterization of NDV in poultry in Kenya. We therefore recommend further studies of the genetic nature of NDV in the Kenyan wild bird population so as to better understand the NDV circulating in wild birds and their relationship with NDV in poultry in Kenya.

## 5. Conclusions

Our results demonstrate that highly velogenic NDVs are circulating in the poultry populations in live bird markets and poultry keeping households. It is clear that the NDVs circulating in poultry in Kenya have low genetic diversity. This implies the need for controlled cross-border movements of poultry and poultry products to avoid introduction of further diverse strains of NDV. In addition, there is need for further investigation into appropriate control strategies that could prevent further divergence of circulating NDVs. Since NDVs in Kenya are genetically similar to those in Uganda, regional effort in controlling the disease would be beneficial.

## Figures and Tables

**Table 1:** Number of birds sampled in live bird markets (LBMs) and backyard poultry farms (BPFs) in different regions of Kenya between 2014 and 2016.

Region	Number and species of poultry sampled in BPFs and in LBMs							
	BPFs					LBMs		
	Chickens	Ducks	Turkeys	Pigeons	Total	Chickens	Ducks	Total
Lake Victoria Basin	288	27	24	21	360	95	4	99
Western Highlands	324	52	30	27	433	122	15	137
Coast	310	57	20	44	431	71	9	80
Eastern	—	—	—	—	—	64	0	64
Nairobi metropolitan	—	—	—	—	—	102	0	102
Total	922	136	74	92	1224	454	28	482

**Table 2:** Newcastle disease virus detection by the molecular assay in backyard poultry farms (BPFs) and live bird markets (LBMs) in different regions of Kenya.

Factor/variable	Poultry farms				Markets			
	Number of samples (*N*)	Number of NDV-positive samples (*n*)	Proportion of positive samples (%)	*p* value	Number of samples (*N*)	Number of NDV-positive samples (*n*)	Proportion of positive samples (%)	*p* value
*Region*								
L. Victoria Basin	**360**	21	5.8 (2.0–10.4)	0.01	99	6	6.1 (2.4–14.3)	0.003
Western Highlands	**433**	8	1.9 (1.1–8.2)	0.257	137	16	11.7 (6.1–19.1)	0.04
Coast	**431**	4	0.9 (0.4–2.5)	Ref.	80	4	5.0 (1.7–15.8)	0.004
Eastern	—	—	—	—	64	4	6.3 (2.1–16.9)	0.01
Nairobi metropolitan	—	—	—	—	102	22	21.6 (14.7–30.6)	Ref.
Total	1224	33	2.7 (1.9–3.8)	0.001	482	52	10.8 (8.2–13.9)	—

**Table 3:** Mean evolutionary distance between the complete nucleotide sequences of the fusion gene of the study sample, NDVs of genotype V, and the LaSota strain.

Evolutionary distance^1^						
Genotype	SS	Vd	Vb	Vc	Va	II
SS	—	[0.005]	[0.010]	[0.010]	[0.012]	[0.018]
Vd^2^	0.031	—	[0.009]	[0.008]	[0.011]	[0.017]
Vb	0.126	0.112	—	[0.005]	[0.007]	[0.015]
Vc	0.105	0.093	0.070	—	[0.007]	[0.014]
Va	0.142	0.127	0.099	0.090	—	[0.017]
II	0.235	0.224	0.213	0.186	0.224	—

Vd (with SS)	—	—	[0.009]	[0.008]	[0.10]	[0.017]
Vb	—	0.115	—	[0.005]	[0.007]	[0.015]
Vc	—	0.097	0.069	—	[0.006]	[0.014]
Va	—	0.130	0.099	0.089	—	[0.016]
II	—	0.226	0.213	0.186	0.224	—

^1^The number of base substitutions per site obtained by averaging all sequence pairs between subgenotypes of genotypes V and II (which are the common vaccine strains). The first half shows the evolutionary distance estimates obtained when the study sample (KE1007/2016: *MG988405*) is not included in subgenotype Vd, and the second half shows evolutionary distances when the study sample is included in subgenotype Vd. In total, 59 sequences were used: subgenotype Vd (*n* = 5), Vb (*n* = 12), Vc (*n* = 9), Va (*n* = 15), genotype II (*n* = 11), and the study sample. Values in square brackets are standard errors calculated by the bootstrap method (1000 replicates). Analysis was conducted using maximum composite likelihood analysis in MEGA 6. A total of 1662 positions were in the final dataset. ^2^Previous NDVs from Uganda classified under a new subgenotype Vd [32].

## Data Availability

The NDV sequences obtained in this study have been deposited in the GenBank database with accession numbers *MG988405* and *KY007043* to *KY007063*.

## Supplementary Materials

Supplementary Materials Table S1: the accession numbers, strain name, year, and country of isolation of reference sequences used for comparison with study sequences. The references are of known genotypes of Newcastle disease virus and are available in GenBank. Figure S2: phylogenetic tree of the nucleotide sequence of the partial fusion (F) gene (374 bp) of the study. Newcastle disease virus (NDV) and reference sequences of NDV from GenBank include representatives of genotype V including Europe viruses, which are only available as 374 bp fusion gene fragments.

## References

[1] OIE (2018). OIE—listed diseases, infections and infestations in force in 2018. http://www.oie.int/animal-health-in-the-world/oie-listed-diseases-2018.

[2] OIE (2015). Newcastle disease: general inf. disease sheets. http://www.oie.int/fileadmin/Home/eng/Media_Center/docs/pdf/Disease_cards/NEWCAS-EN.pdf.

[3] Amarasinghe G. K., Bejerman N., Kim-Blasdell B. R. (2017). Taxonomy of the order *Mononegavirales*: update 2017. *Archives of Virology*.

[4] Ganar K., Das M., Sinha S., Kumar S. (2014). Newcastle disease virus: current status and our understanding. *Virus Research*.

[5] Gogoi P., Ganar K., Kumar S. (2015). Avian paramyxovirus: a brief review. *Transboundary and Emerging Diseases*.

[6] Martín-García F., Mendieta-Moreno J. I., Mendieta J., Gómez-Puertas P. (2012). Molecular dynamics analysis of conformational change of paramyxovirus F protein during the initial steps of membrane fusion. *Biochemical and Biophysical Research Communications*.

[7] Kim S.-H., Wanasen N., Paldurai A., Xiao S., Collins P. L., Samal S. K. (2013). Newcastle disease virus fusion protein is the major contributor to protective immunity of genotype-matched vaccine. *PLoS One*.

[8] Fan S., Wang T., Gao X. (2015). Phylogenetic analysis of Newcastle disease viruses isolated from wild birds in the Poyang Lake region of China. *Journal of Veterinary Medical Science*.

[9] Diel D. G., da Silva L. H. A., Liu H., Wang Z., Miller P. J., Afonso C. L. (2012). Genetic diversity of avian paramyxovirus type 1: proposal for a unified nomenclature and classification system of Newcastle disease virus genotypes. *Infection, Genetics and Evolution*.

[10] de Almeida R. S., Hammoumi S., Gil P. (2013). New avian paramyxoviruses type I strains identified in Africa provide new outcomes for phylogeny reconstruction and genotype classification. *PLoS One*.

[11] Susta L., Jones M. E. B., Cattoli G. (2015). Pathologic characterization of genotypes XIV and XVII Newcastle disease viruses and efficacy of classical vaccination on specific pathogen-free birds. *Veterinary Pathology*.

[12] Dimitrov K. M., Ramey A. M., Qiu X., Bahl J., Afonso C. L. (2016). Temporal, geographic, and host distribution of avian paramyxovirus 1 (Newcastle disease virus). *Infection, Genetics and Evolution*.

[13] Damena D., Fusaro A., Sombo M. (2016). Characterization of Newcastle disease virus isolates obtained from outbreak cases in commercial chickens and wild pigeons in Ethiopia. *SpringerPlus*.

[14] Molini U., Aikukutu G., Khaiseb S., Cattoli G., Dundon W. G. (2017). First genetic characterization of newcastle disease viruses from Namibia: identification of a novel VIIk subgenotype. *Archives of Virology*.

[15] Snoeck C. J., Owoade A. A., Couacy-Hymann E. (2013). High genetic diversity of Newcastle disease virus in poultry in West and Central Africa: cocirculation of genotype XIV and newly defined genotypes XVII and XVIII. *Journal of Clinical Microbiology*.

[16] ACIAR (May 2018). Newcastle disease control in Africa. http://aciar.gov.au/files/ias_87-web.pdf.

[17] Copland J. W., Alders R. G. (2013). The Australian village poultry development programme in Asia and Africa. *World’s Poultry Science Journal*.

[18] Njagi L. W., Nyaga P. N., Mbuthia P. G., Bebora L. C., Michieka J. N., Minga U. M. (2010). A retrospective study of factors associated with Newcastle disease outbreaks in village indigenous chickens, in Africa. *Bulletin of Animal Health and Production in Africa*.

[19] Adeniyi O. R., Oguntunji A. O. (2011). A socio-economic survey of cultural practices and management of village poultry production in Ondo area, Nigeria. *Livestock Research for Rural Development*.

[20] Kenya National Bureau of Statistics (June 2018). Economic survey 2016. http://www.knbs.or.ke.

[21] Charan J., Kantharia N. (2013). How to calculate sample size in animal studies?. *Journal of Pharmacology and Pharmacotherapeutics*.

[22] Njagi L. W., Nyaga P. N., Mbuthia P. G. (2010). Prevalence of Newcastle disease virus in village indigenous chickens in varied agro-ecological zones in Kenya. *Livestock Research for Rural Development*.

[23] OIE (2013). *Manual of Diagnostic Tests and Vaccines for Terrestrial Animals*.

[24] Liu H., Wang Z., Wu Y. (2007). Molecular epidemiological analysis of Newcastle disease virus isolated in China in 2005. *Journal of Virological Methods*.

[25] Ye J., Coulouris G., Zaretskaya I., Cutcutache I., Rozen S., Madden T. L. (2012). Primer-BLAST: a tool to design target-specific primers for polymerase chain reaction. *BMC Bioinformatics*.

[26] Shabbir M. Z., Zohari S., Yaqub T. (2013). Genetic diversity of Newcastle disease virus in Pakistan: a countrywide perspective. *Virology Journal*.

[27] Mohamed M. H. A., Kumar S., Paldurai A., Samal S. K. (2011). Sequence analysis of fusion protein gene of Newcastle disease virus isolated from outbreaks in Egypt during 2006. *Virology Journal*.

[28] Rozas J., Ferrer-Mata A., Sánchez-DelBarrio J. C. (2017). DnaSP 6: DNA sequence polymorphism analysis of large datasets. *Molecular Biology and Evolution*.

[29] Benson D. A., Cavanaugh M., Clark K. (2017). GenBank. *Nucleic Acids Research*.

[30] Edgar R. C., Batzoglou S. (2006). Multiple sequence alignment. *Current Opinion in Structural Biology*.

[31] Tamura K., Stecher G., Peterson D., Filipski A., Kumar S. (2013). MEGA6: molecular evolutionary genetics analysis version 6.0. *Molecular Biology and Evolution*.

[32] Byarugaba D. K., Mugimba K. K., Omony J. B. (2014). High pathogenicity and low genetic evolution of avian paramyxovirus type I (Newcastle disease virus) isolated from live bird markets in Uganda. *Virology Journal*.

[33] McCarron M., Munyua P., Cheng P. Y. (2015). Understanding the poultry trade network in Kenya: implications for regional disease prevention and control. *Preventive Veterinary Medicine*.

[34] Chaka H., Goutard F., Gil P. (2013). Serological and molecular investigation of Newcastle disease in household chicken flocks and associated markets in Eastern Shewa zone, Ethiopia. *Tropical Animal Health and Production*.

[35] Susta L., Hamal K. R., Miller P. J. (2014). Separate evolution of virulent Newcastle disease viruses from Mexico and Central America. *Journal of Clinical Microbiology*.

[36] Rue C. A., Susta L., Brown C. C. (2010). Evolutionary changes affecting rapid identification of 2008 Newcastle disease viruses isolated from double-crested cormorants. *Journal of Clinical Microbiology*.

[37] Wehmann E., Ujvári D., Mazija H. (2003). Genetic analysis of Newcastle disease virus strains isolated in Bosnia-Herzegovina, Croatia, Slovenia and Yugoslavia, reveals the presence of only a single genotype, V, between 1979 and 2002. *Veterinary Microbiology*.

[38] Diel D. G., Miller P. J., Wolf P. C. (2012). Characterization of Newcastle disease viruses isolated from cormorant and gull species in the United States in 2010. *Avian Diseases*.

[39] Cardenas Garcia S., Navarro Lopez R., Morales R. (2013). Molecular epidemiology of Newcastle disease in Mexico and the potential spillover of viruses from poultry into wild bird species. *Applied and Environmental Microbiology*.

[40] Fernandes C. C., Varani A. M., Lemos E. G. M. (2014). Molecular and phylogenetic characterization based on the complete genome of a virulent pathotype of Newcastle disease virus isolated in the 1970s in Brazil. *Infection, Genetics and Evolution*.

[41] Wehmann E., Czeglédi A., Werner O., Kaleta E. F., Lomniczi B. (2003). Occurrence of genotypes IV, V, VI and VIIa in Newcastle disease outbreaks in Germany between 1939 and 1995. *Avian Pathology*.

[42] Czegledi A., Herczeg J., Hadjiev G., Doumanova L., Wehmann E., Lomniczi B. (2002). The occurrence of five major Newcastle disease virus genotypes (II, IV, V, VI and VIIb) in Bulgaria between 1959 and 1996. *Epidemiology and Infection*.

