# Supplementary material for: Molecular Characterization of Newcastle Disease Virus from Backyard Poultry Farms and Live Bird Markets in Kenya
Source: Int J Microbiol. 2018 Aug 5;2018:2368597. doi: 10.1155/2018/2368597 (PMC6098844; doi:10.1155/2018/2368597)
Supplement: Supplementary Materials — Table S1: the accession numbers, strain name, year, and country of isolation of reference sequences used for comparison with study sequences. The references are of known genotypes of Newcastle disease virus and are available in GenBank. Figure S2: phylogenetic tree of the nucleotide sequence of the partial fusion (F) gene (374 bp) of the study. Newcastle disease virus (NDV) and reference sequences of NDV from GenBank include representatives of genotype V including Europe viruses, which are only available as 374 bp fusion gene fragments. [file 2368597.f1.pdf]

Table S1: The Fusion gene reference sequences from GenBank and study sequences used in analysis

Accession No	Species	Country of origin	Isolate name	Year of collection	Genotype
AB465607	Chicken	Japan	Japan/Ishi/62	1962	I
Z12110	Chicken	Ireland	Ulster Mutant	1967	I
AY935492	Chicken	Australia	98-1249	1998	I
AY935499	Chicken	Australia	I-2vaccine	2005	I
AY935500	Chicken	Australia	I-2progenitor	2005	I
FJ600539	Mule duck	China	FJ0801	2008	I
HM063424	Water rail	China	R8/Guang-dong	2005	I
AF077761	Chicken	USA	Lasota	1946	II
AF309418		USA	B1	1947	II
AY225110		China	HB92/V4		II
EU289028	Turkey	USA	VG/GA	1987	II
EF201805		---	Mukteswar	1940	III
EF211808	Goose	China	JS/2/05/Go	1940	III
EF430159	Chicken	China	JS/7/05Ch	1940	III
EF589136	Fowl	China	Guizhou	1940	III
FJ480786	Mallard	China	NDV/Mallard/HLJ383/06	1940	III
AY741404		UK	Herts/33	1940	IV
EU293914		China	Italien	1940	IV
GU187941	Chicken	India	NDV-2//Namakkal/ Tamil Nadu	1940	IV
M24702		UK	Herts33	1940	IV
AF163440		China	F48E9		IX
AY341061	Chicken	China	LuoY		IX
FJ436302	Chicken	China	F48E8	1946	IX
FJ436304	Chicken	China	FJ01/85	1985	IX
FJ436306	Duck	China	JS/1/02	2002	IX
FJ705459	Cormorant	Canada	Cormorant/98CNN3-V1125/1998	1998	V a
FJ705460	Cormorant	Canada	Cormorant/95DC02150/1995	1995	V a
FJ705461	Cormorant	Canada	Cormorant/95DC2345/1995	1995	V a
JN872161	Cormorant	USA	Cormorant/Wisconsin/498260-2/07	1997	V a
AY288993	Chicken	Honduras	Chicken/Honduras/15/00	2000	V b
AY562987	Fowl	USA	Gamefowl/U.S.(CA)/211472/02	2002	V b
AY562990	Psittacine	USA	Largo/71	1971	V b
EF520718	Gamefowl	USA	Gamefowl/US (CA)/212676/2002	2002	V b
JN872181	Chicken	Honduras	Chicken/Honduras/44813/2000	2000	V b
JN942027	Chicken	Nicaragua	Fighting Cock/Nicaragua/95066-9/2001	2001	V b
JN942039	Amazon parrot	USA	Amazon/California/28936/1988	1988	V b
EU518683	Chicken	Mexico	Chicken/Mexico (Estado de Mexico)/465/2005	2005	V c
KJ577136	Chicken	Mexico	Chimalhuacan	1973	V c
HM117720	Chicken	Mexico	NDV-P05	2005	V c
JQ697743	Chicken	Mexico	Chicken/MX/NC02-634/2010	2010	V c
JQ697744	Chicken	Mexico	Chicken/MX/NC04-635/2010	2010	V c
JX974435	Chicken	Mexico	NDV1/10	2010	V c
KC808508	Gamefowl	Mexico	Gamefowl/Mex/616/2008	2008	V c
KC808509	Gamefowl	Mexico	Gamefowl/Mex(DF)/619/2008	2008	V c
KC808510	Scarlet macaw	Mexico	Scarlet macaw/Mex(Chiapas)/672-ZM12/2009	2009	V c
AF218134	Pheasant	Italy	IT-47	1974	Va
AF218136	Turkey	Italy	IT-51B	1972	Va
AF525370	Chicken	Germany	DE-173	1970	Va
AF525375	Chicken	Germany	DE-183	1974	Va
AF525377	Amazon	Germany	DE-190	1970	Va
AF525396		Yugoslovia	Yu(Vo)-1744	2002	Va
AY116971		Slovonija	HR-448	1979	Va
AY444496	Yellow nape parrot	USA	U.S(MA)/19120/87	1987	Va
EF065682	Chicken	USA	rAnhinga		Va

GQ288382	Cormorant	Canada	Cormorant/98 CNN3-V1125DC02150/1998	1998	Va
GQ288383	Cormorant	Canada	Cormorant/Canada/95DC02150/1995	1995	Va
GQ288387	Cormorant	USA (MN)	Cormorant/US (MN) 92-40140/1992	1992	Va
GQ288388	Cormorant	USA (CA)	Cormorant/US(CA)/92-23071/1992	1992	Va
AF402118		Bulgaria	BG-55	1975	Va
HG937573	Chicken	Uganda	NDV/chicken/Uganda/MU024/2011	2011	Vd
HG937580	Chicken	Uganda	NDV/chicken/Uganda/MU040/2011	2011	Vd
JQ217418	Chicken	Kenya	A89	2010	Vd
JQ217419	Chicken	Kenya	A48	2010	Vd
JQ217420	Chicken	Kenya	A148	2010	Vd
KY007043	Chicken	Kenya(Nairobi)	NDV/KE0576/2015	2015	Study sequence
KY007044	Chicken	Kenya(Nairobi)	NDV/KE0733/2015	2015	Study sequence
KY007045	Chicken	Kenya Makueni)	NDV/KE0679/2015	2015	Study sequence
KY007046	Chicken	Kenya (Bomet)	NDV/KE0673/2015	2015	Study sequence
KY007047	Chicken	Kenya (Kitui)	NDV/KE0601/2015	2015	Study sequence
KY007048	Chicken	Kenya (Bungoma)	NDV/KE1002/2016	2016	Study sequence
KY007049	Chicken	Kenya (Makueni)	NDV/KE0678/2015	2015	Study sequence
KY007050	Chicken	Kenya (Bungoma)	NDV/KE0697/2015	2015	Study sequence
KY007051	Chicken	Kenya (Homabay)	NDV/KE0695/2015	2015	Study sequence
KY007052	Chicken	Kenya (Homabay)	NDV/KE0687/2015	2015	Study sequence
KY007053	Chicken	Kenya (Makueni)	NDV/KE0676/2015	2015	Study sequence
KY007054	Chicken	Kenya (Busia)	NDV/KE2055/2016	2016	Study sequence
KY007055	Chicken	Kenya (Busia)	NDV/KE2054/2015	2015	Study sequence
KY007056	Chicken	Kenya (Migori)	NDV/KE0715/2015	2015	Study sequence
KY007057	Chicken	Kenya (Bungoma)	NDV/KE1004/2016	2016	Study sequence
KY007058	Chicken	Kenya (Kitengela)	NDV/KE0525/2015	2015	Study sequence
KY007059	Chicken	Kenya (Kilifi)	NDV/KE0410/2015	2015	Study sequence
KY007060	Chicken	Kenya (Machakos)	NDV/KE0647/2015	2015	Study sequence
KY007061	Chicken	Kenya (Nairobi)	NDV/KE0638/2015	2015	Study sequence
KY007062	Chicken	Kenya (Malaba)	NDV/KE0660/2015	2015	Study sequence
KY007063	Chicken	Kenya (Kitengela)	NDV/KE0523/2015	2015	Study sequence
MG988405	Chicken	Kenya (Busia)	NDV/KE1007/2016	2016	Study sequence
AY288995	Dove	Italy	Dove/Italy/2736/00	2000	VI
AY288996	Pigeon	Italy	Pigeon/Italy/1166/00	2000	VI
AY288997	Chicken	Kenya	Chicken/Kenya/139/90	1990	VI
FJ865434	Pigeon	China	S-1	2002	VI
GQ507801	Chicken	South Korea	Kr-102/89	1989	VI
JX518532	laughing Dove	Kenya	Laughing Dove/B2/Kenya/Isiolo	2012	VI
AY444497		Indonesia	Moluccan/Indonesia/904/87	1987	VII
GQ288381	Cormorant	USA (CA)	Cormorant/US(CA)/D9704285/1997	1997	VII
AF358786	Chicken	Taiwan	TW/2000	2000	VII
AF458010	Chicken	China	JS-3/00	2000	VII
GQ245818	Ostrich	China	YZ-22-07-Os	2007	VII
GU227738	Dove	Serbia	NDV/Serbia/749/2007	2007	VII
AY734534	Chicken	Argentina	Trenque Lauquen	1970	VIII
FJ751918	Chicken	China	QH1	1979	VIII
FJ751919	Chicken	China	QH4	1985	VIII
FJ705464	Mallard	USA	Mallard/US(OH)/04-411/2004	2004	X
FJ705467	Mallard	USA	Mallard/US(MN)/MN00-32/2000	2000	X
FJ705468	Mottled duck	USA	Mottled duck/US(TX)/TX01-130/2001	2001	X
HQ266602	Chicken	Madagascar	MG/725/08	2008	XI
HQ266603	Chicken	Madagascar	MG/1992/08	2008	XI
HQ266604	Chicken	Madagascar	MG/Meola/08	2008	XI
JN627504	goose	China	Goose/China-GD/12/2011	2011	XII
JN627506	Goose	China	Goose/China-GD/20/2011	2011	XII
JN627507	goose	China	Goose/China-GD/1003/2010	2010	XII
JN627508	goose	China	Goose/China-GD/450/2011	2011	XII
JN800306	Chicken	Peru	Poultry/Peru/1918-03/2008	2008	XII
AY865652	*Sterna albifrons*	Russia	Sterna/Astr/2755/2001	2001	XIII
GU182323	Chicken	Pakistan	SPVC/Karachi/NDV/43/2008	2008	XIII
GU182331	Chicken	Pakistan	SPVC/Karachi/NDV/33/2007	2007	XIII

JN682190	Chicken	Pakistan	Chicken/CP/Islamabad2/2010	2010	XIII
HF969214	Chicken	Nigeria	Chicken/Nigeria/NIE10-333/2011	2011	XIV
JX119193	Chicken	Dominican Republic	DominicanRepublic//499-31/2008	2008	XVI
JX186997	Chicken	Dominican Republic	Dominican/Republic/867/2008	2008	XVI
JX915242	Chicken	Dominican Republic	DominicanRepublic/28138-4/1986	1986	XVI
JX915243	Chicken	Mexico	Mexico/Queretaro/452/1947	1947	XVI
HF969184	Chicken	Ivory Coast	CIV08-103	2007	XVII
FJ772446		Nigeria	Avian/913-1/Nigeria/2006	2006	XVII
FJ772449		Nigeria	Avian/913-33/Nigeria/2006	2006	XVII
FJ772455		Mauritania	Avian/1532/14/Mauritania/2006	2006	XVIII
FJ772466	Chicken	IvoryCoast	Chicken/2601/Ivory Coast/2008	2008	XVIII
HF969127	Chicken	Ivory Coast	CIV08-069	2007	XVIII
EF612277		USA	AK/196	1998	Class I

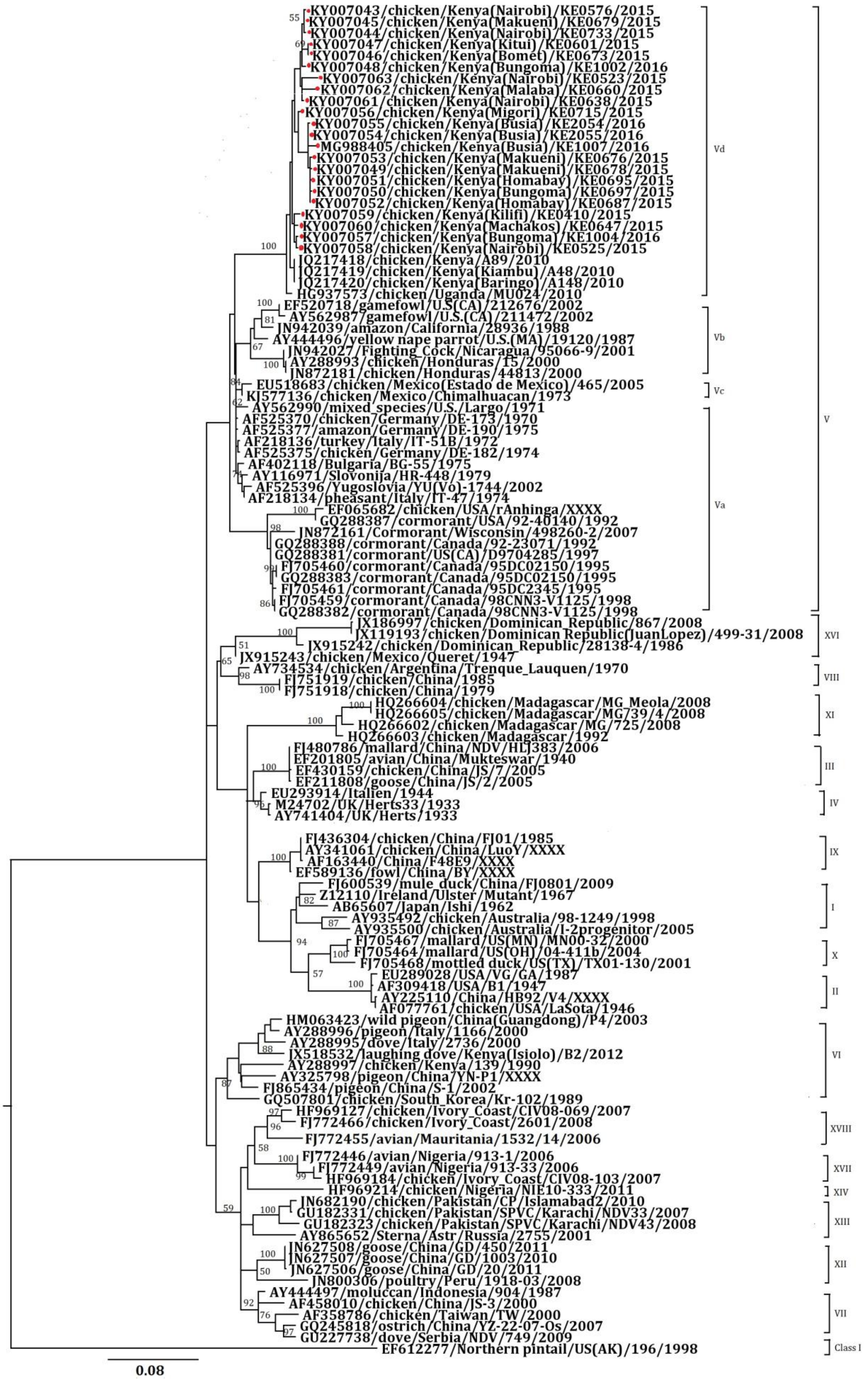
KY007043/chicken/Kenya(Nairobi)/KE0576/2015
KY007045/chicken/Kenya(Makueni)/KE0679/2015
KY007044/chicken/Kenya(Nairobi)/KE0733/2015
KY007047/chicken/Kenya(Kitui)/KE0601/2015
KY007046/chicken/Kenya(Bomet)/KE0673/2015
KY007048/chicken/Kenya(Bungoma)/KE1002/2016
KY007063/chicken/Kenya(Nairobi)/KE0523/2015
KY007062/chicken/Kenya(Malaba)/KE0660/2015
KY007061/chicken/Kenya(Nairobi)/KE0638/2015
KY007056/chicken/Kenya(Migori)/KE0715/2015
KY007055/chicken/Kenya(Busia)/KE2054/2016
KY007054/chicken/Kenya(Busia)/KE2055/2016
MG988405/chicken/Kenya(Busia)/KE1007/2016
KY007053/chicken/Kenya(Makueni)/KE0676/2015
KY007049/chicken/Kenya(Makueni)/KE0678/2015
KY007051/chicken/Kenya(Homabay)/KE0695/2015
KY007050/chicken/Kenya(Bungoma)/KE0697/2015
KY007052/chicken/Kenya(Homabay)/KE0687/2015
KY007059/chicken/Kenya(Kilifi)/KE0410/2015
KY007060/chicken/Kenya(Machakos)/KE0647/2015
KY007057/chicken/Kenya(Bungoma)/KE1004/2016
KY007058/chicken/Kenya(Nairobi)/KE0525/2015
JQ217418/chicken/Kenya/A89/2010
JQ217419/chicken/Kenya(Kiambu)/A48/2010
JQ217420/chicken/Kenya(Baringo)/A148/2010
HG937573/chicken/Uganda/MU024/2010
Vd
EF520718/gamefowl/U.S(CA)/212676/2002
AY562987/gamefowl/U.S.(CA)/211472/2002
JN942039/amazon/California/28936/1988
AY444496/yellow nape parrot/U.S.(MA)/19120/1987
JN942027/Fighting_Cock/Nicaragua/95066-9/2001
AY288993/chicken/Honduras/15/2000
JN872181/chicken/Honduras/44813/2000
Vb
EU518683/chicken/Mexico(Estado de Mexico)/465/2005
KJ577136/chicken/Mexico/Chimalhuacan/1973
Vc
AY562990/mixed_species/U.S./Largo/1971
AF525370/chicken/Germany/DE-173/1970
AF525377/amazon/Germany/DE-190/1975
AF218136/turkey/Italy/IT-51B/1972
AF525375/chicken/Germany/DE-182/1974
AF402118/Bulgaria/BG-55/1975
AY116971/Slovonija/HR-448/1979
AF525396/Yugoslovia/YU(Vo)-1744/2002
AF218134/pheasant/Italy/IT-47/1974
EF065682/chicken/USA/rAnhinga/XXXX
GQ288387/cormorant/USA/92-40140/1992
JN872161/Cormorant/Wisconsin/498260-2/2007
GQ288388/cormorant/Canada/92-23071/1992
GQ288381/cormorant/US(CA)/D9704285/1997
FJ705460/cormorant/Canada/95DC02150/1995
GQ288383/cormorant/Canada/95DC02150/1995
FJ705461/cormorant/Canada/95DC2345/1995
FJ705459/cormorant/Canada/98CNN3-V1125/1998
GQ288382/cormorant/Canada/98CNN3-V1125/1998
Va
V
JX186997/chicken/Dominican_Republic/867/2008
JX119193/chicken/Dominican Republic(JuanLopez)/499-31/2008
JX915242/chicken/Dominican_Republic/28138-4/1986
JX915243/chicken/Mexico/Queret/1947
XVI
AY734534/chicken/Argentina/Trenque_Lauquen/1970
FJ751919/chicken/China/1985
FJ751918/chicken/China/1979
VIII
HQ266604/chicken/Madagascar/MG_Meola/2008
HQ266605/chicken/Madagascar/MG739/4/2008
HQ266602/chicken/Madagascar/MG/725/2008
HQ266603/chicken/Madagascar/1992
XI
FJ480786/mallard/China/NDV/HLJ383/2006
EF201805/avian/China/Mukteswar/1940
EF430159/chicken/China/JS/7/2005
EF211808/goose/China/JS/2/2005
III
EU293914/Italien/1944
M24702/UK/Herts33/1933
AY741404/UK/Herts/1933
IV
FJ436304/chicken/China/FJ01/1985
AY341061/chicken/China/LuoY/XXXX
AF163440/China/F48E9/XXXX
EF589136/fowl/China/BY/XXXX
IX
FJ600539/mule_duck/China/FJ0801/2009
Z12110/Ireland/Ulster/Mutant/1967
AB65607/Japan/Ishi/1962
AY935492/chicken/Australia/98-1249/1998
AY935500/chicken/Australia/I-2progenitor/2005
I
FJ705467/mallard/US(MN)/MN00-32/2000
FJ705464/mallard/US(OH)/04-411b/2004
FJ705468/mottled duck/US(TX)/TX01-130/2001
X
EU289028/USA/VG/GA/1987
AF309418/USA/B1/1947
AY225110/China/HB92/V4/XXXX
AF077761/chicken/USA/LaSota/1946
II
HM063423/wild pigeon/China(Guangdong)/P4/2003
AY288996/pigeon/Italy/1166/2000
AY288995/dove/Italy/2736/2000
JX518532/laughing dove/Kenya(Isiolo)/B2/2012
AY288997/chicken/Kenya/139/1990
AY325798/pigeon/China/YN-P1/XXXX
FJ865434/pigeon/China/S-1/2002
GQ507801/chicken/South_Korea/Kr-102/1989
VI
HF969127/chicken/Ivory_Coast/CIV08-069/2007
FJ772466/chicken/Ivory_Coast/2601/2008
FJ772455/avian/Mauritania/1532/14/2006
XVIII
FJ772446/avian/Nigeria/913-1/2006
FJ772449/avian/Nigeria/913-33/2006
HF969184/chicken/Ivory_Coast/CIV08-103/2007
XVII
HF969214/chicken/Nigeria/NIE10-333/2011
XIV
JN682190/chicken/Pakistan/CP/Islamabad2/2010
GU182331/chicken/Pakistan/SPVC/Karachi/NDV33/2007
GU182323/chicken/Pakistan/SPVC/Karachi/NDV43/2008
AY865652/Sterna/Astr/Russia/2755/2001
XIII
JN627508/goose/China/GD/450/2011
JN627507/goose/China/GD/1003/2010
JN627506/goose/China/GD/20/2011
JN800306/poultry/Peru/1918-03/2008
XII
AY444497/moluccan/Indonesia/904/1987
AF458010/chicken/China/JS-3/2000
AF358786/chicken/Taiwan/TW/2000
GQ245818/ostrich/China/YZ-22-07-Os/2007
GU227738/dove/Serbia/NDV/749/2009
VII
EF612277/Northern pintail/US(AK)/196/1998
Class I
0.08

Figure S2: Phylogenetic tree of nucleotide sequence of the partial Fusion (F) gene (374bp) of Newcastle disease virus (NDV) in the study (● marked) and reference sequences for NDV from GenBank including representatives of genotype V from various parts of the world (including Europe). The evolutionary history was inferred by using the Maximum Likelihood method based on the Kimura-2 parameter. The analysis involved 119 nucleotide sequences. The tree is rooted. The analyses were conducted in MEGA
